# Enhancing patient‐specific quality assurance for VMAT for breast cancer treatment: A machine learning approach for gamma passing rate (GPR) prediction

**DOI:** 10.1002/acm2.70251

**Published:** 2025-09-08

**Authors:** Francis C. Djoumessi Zamo, Anthony Colliaux, Valérie Blot‐Lafond, Ndontchueng Moyo, Christopher F Njeh

**Affiliations:** ^1^ Centre Clinical Soyaux Centre de Radiothérapie Angouleme Saint Michel France; ^2^ Centre de Physique Atomique Moléculaire et Optique University of DOUALA Douala Cameroun; ^3^ Radiation Oncology Department School of Medicine Indiana University Indianapolis Indiana USA

## Abstract

**Background:**

Modern radiation therapy for breast cancer has significantly advanced with the adoption of volumetric modulated arc therapy (VMAT), offering enhanced precision and improved treatment efficiency.

**Purpose:**

To ensure the accuracy and precision of such complex treatments, a robust patient‐specific quality assurance (PSQA) protocol is essential. This study investigates the potential of machine learning (ML) models to predict gamma passing rates (GPR), a key metric in PSQA.

**Methods:**

A dataset comprising 863 VMAT plans was used to develop and compare seven ML models: Histogram‐based gradient boosting regressor, random forest regressor, extra trees regressor, gradient boosting regressor, linear regression, AdaBoost regressor, and Multi‐layer perceptron regressor. These models incorporated anatomical, dosimetric, and plan complexity features.

**Results:**

Among the evaluated models, the extra trees regressor (ETR), random forest regressor (RFR), and gradient boosting regressor (GBR) demonstrated the best performance, achieving mean absolute errors (MAEs) of 0.51%, 0.52%, and 0.51%, and mean squared errors (MSEs) of 0.0051%, 0.0051%, and 0.0052%, respectively, on the validation dataset.

**Conclusions:**

This study highlights the promise of ML‐based approaches in streamlining PSQA processes, thereby supporting the quality assurance of breast cancer treatments using VMAT.

## 1 INTRODUCTION

Volumetric modulated arc therapy (VMAT) has become a cornerstone in breast cancer radiotherapy, offering conformal dose delivery with high precision while minimizing exposure to surrounding healthy tissues.^1^, ^2^, ^3^, ^4^ Because of the inherent complexity of VMAT plans—often involving multiple arcs and dynamic multi‐leaf collimator (MLC) movements—robust patient‐specific quality assurance (PSQA) is essential to ensure treatment accuracy.^5^

A key component of PSQA is the gamma passing rate (GPR), which quantifies the agreement between planned and delivered dose distributions using criteria based on dose difference and distance‐to‐agreement.^6^ Despite its importance, traditional PSQA methods are time‐consuming and resource‐intensive, typically requiring physical measurements with ionization chambers, detector arrays, films, or electronic portal imaging devices (EPIDs).^7^, ^8^ These procedures can create workflow bottlenecks, particularly in high‐throughput clinical settings.^9^

Recent advances in machine learning (ML) have shown promise in various aspects of radiotherapy, including treatment planning, outcome prediction, and quality assurance.^10^, ^11^, ^12^, ^13^, ^14^ ML models have been explored for predicting PSQA outcomes, potentially reducing the need for routine measurements by identifying plans likely to pass or fail based on plan characteristics.^15^ However, to date, only a few studies—such as those by Noblet et al.^16^ and Boutry et al.—^17^ have applied ML or deep learning (DL) to PSQA in breast cancer, and these have primarily focused on classification tasks.

To our knowledge, no prior study has addressed the prediction of continuous GPR values for breast cancer patients treated with VMAT. This study aims to fill that gap by leveraging ML to predict GPRs, thereby streamlining the PSQA process. Using a dataset of 863 VMAT plans, we developed and compared seven ML models incorporating anatomical, dosimetric, and plan complexity features. We evaluated their performance using relevant regression metrics. Our findings suggest that ML‐based prediction of GPR can significantly reduce the PSQA workload while maintaining high standards of treatment quality and patient safety.

## 2 MATERIALS AND METHODS

### 2.1 Data collection

A retrospective dataset of 863 VMAT plans for breast cancer treatments was collected from patients previously treated at our facility. All breast cancer cases were included, regardless of tumor type or prescribed dose, to ensure comprehensive representation of departmental treatments. Patient data were anonymized to protect privacy, and an institutional review board (IRB) exemption was granted due to the retrospective nature of the study.

All treatment plans were created using the Philips Pinnacle planning system (version 16.3, Philips Healthcare) and delivered on one of three Elekta linear accelerators (Elekta AB), each equipped with a 160‐leaf Agility collimator. Treatments were delivered using VMAT with 6 MV photon beams.

#### 2.1.1 Patient‐Specific quality assurance data: Model target

In accordance with departmental policy, all 863 treatment plans underwent PSQA prior to delivery. PSQA was performed using the Delta4 biplanar diode detector system (ScandiDos), and results were evaluated using GPR analysis. The evaluation criteria included a 3%/3 mm threshold with a local dose threshold of 20%. These GPR values were compiled in an Excel file and served as the target variable for model prediction.

#### 2.1.2 Model features

The input features were categorized into two groups:

**a. Anatomical data**:

Extracted from treatment planning CT scans and structure contours:

1. Presence of a boost volume in the treated breast
2. Laterality of the treated breast (Right, Left, Bilateral)
3. Inclusion of internal mammary chain (IMC) in the treatment
4. Inclusion of supraclavicular node (Clav_Node)
5. Heart volume
6. Planning target volume (PTV) of the breast

**FIGURE 1:**
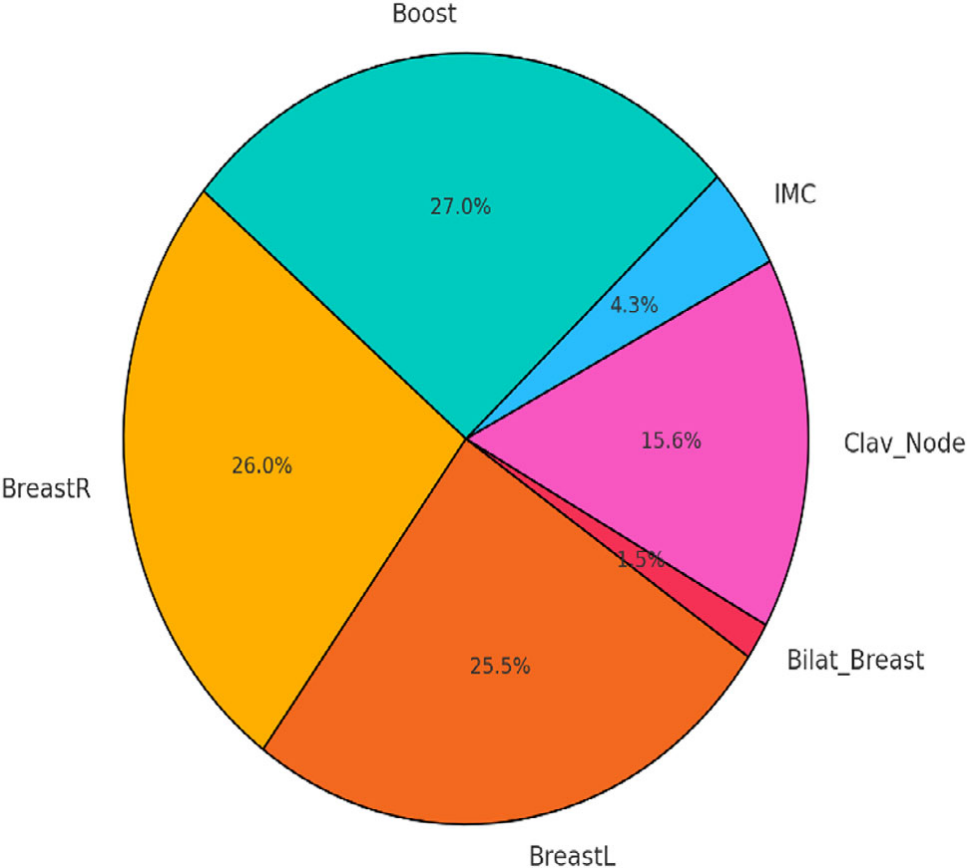
Distribution of treatment sites and Boost cases for the plans of this study.

A pie chart illustrating the distribution of treatment sites is shown in Figure 1.

**b. Radiotherapy plan features**:

Using the UCOMX software developed by Cavinato et al.,^18^ dosimetric and plan complexity metrics were extracted from the treatment planning system. These features are summarized in Table 1.

**TABLE 1:** List of all the metrics and parameters used to build the seven machine learning regression models.

Number	Metrics	References
1	MUs: Total number of monitor units delivered	[19, 20, 21]
2	MUCA: Average number of MU delivered per control arc	
3	MD: Modulation degree	[22]
4	LT: Average total leaf travel per control point	[21]
5	LTNL: Average leaf travel per involved leaf	
6	MUdeg: Monitor unit per degree of gantry rotation	[23]
7	MCSv: Modulation complexity score	[21]
8	AAV: Aperture area variability	[24]
9	PI: Plan irregularity	[21]
10	PM: Plan modulation	[21]
11	PA: Average beam area	[21]
12	ALG: Average leaf gap	[25]
13	Psmall_20 mm: Percentage of BEVs with EFS <20 mm	
14	Psmall_30 mm: Percentage of BEVs with EFS <30 mm	
15	EFS: Equivalent field size	[26]
16	SAS10 mm: Fraction of gaps <10 mm	[27, 28]
17	SAS25 mm: Fraction of gaps <25 mm	
18	SAS50 mm: Fraction of gaps <50 mm	
19	EM: Edge metric	[29]
20	BJAR: Average BEV‐jaws area ratio	
21	P: Average BEV's perimeter	
22	NL: Average number of involved leaves	
23	AL: Average arc length	
24	GT: Total angular distance travelled by the gantry	

### 2.2 Machine learning model development and validation

#### 2.2.1 Regression models

Seven machine learning regression models were developed and evaluated:

1. Histogram‐based gradient boosting regressor (HGBR)
2. Random forest regressor (RFR)
3. Extra trees regressor (ETR)
4. Gradient boosting regressor (GBR)
5. Linear regression (LR)
6. AdaBoost regressor (ABR)
7. Multi‐layer perceptron regressor (MLPR)

All models were implemented using Python and the Scikit‐learn library.^30^

#### 2.2.2 Model training and validation

**a. Training process**:

To mitigate the influence of outliers, the interquartile range (IQR) method was applied, with a cutoff set at three times the IQR for GPR values. The dataset was split into training (80%) and testing (20%) subsets.

To address potential imbalance in the GPR distribution, random oversampling was applied to the minority class in the training set, enhancing model sensitivity to underrepresented data.

Feature scaling was performed using the StandardScaler to ensure uniform contribution across features. Hyperparameter tuning was conducted using GridSearchCV, which systematically evaluates combinations of parameters to identify the optimal configuration.

All models underwent 10‐fold cross‐validation to assess generalizability and reduce overfitting.

**b. Validation metrics**

Model performance was evaluated using:

1. Mean absolute error (MAE)
2. Mean squared error (MSE)
3. Root mean squared error (RMSE)

These metrics were computed for both training and validation datasets to assess predictive accuracy and generalization capability.

## 3 RESULTS

### 3.1 Visualization of GPR trends

**FIGURE 2:**
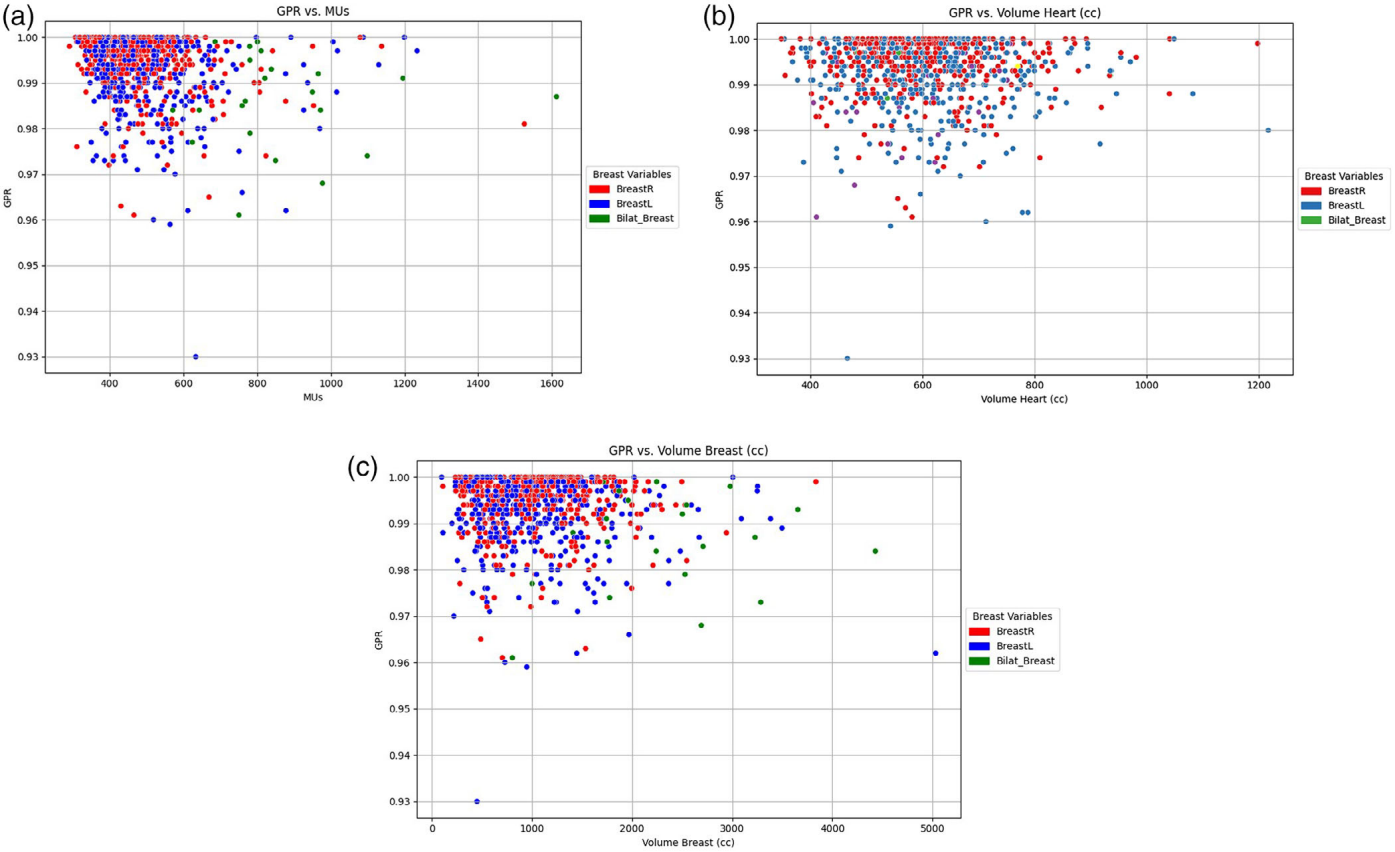
Illustration of gamma pass rate (GPR) trends against (a) monitor units (Mus), (b) volume heart and (c) volume breast for different treatment sites (BreastR, BreastL, and Bilat_Breast).

To explore the relationship between GPR and key treatment parameters, scatter plots were generated for GPR versus Monitor Units (MUs), heart volume, and breast volume. These visualizations are presented in Figure 2, illustrating clustering patterns and variability across different treatment characteristics.

Figure 2a reveals that higher MUs do not necessarily correlate with improved GPR values. Although most data points cluster around high GPR values (>0.98), increased variability is observed as MUs exceed 800. This suggests that more complex treatment plans requiring higher MUs may warrant additional scrutiny during PSQA, as also noted by Du et al.^19^

In examining heart volume (Figure 2b), GPR remains consistently high (>0.96) across most cases. However, slight deviations are observed in plans involving heart volumes greater than 600 cc, indicating the importance of optimizing dose constraints in these scenarios to maintain treatment safety.

The distribution of GPR values becomes broader as breast volume increases (Figure 2c). Although most plans still achieve high GPR, volumes exceeding 3000 cc appear to introduce additional complexity, potentially necessitating customized QA strategies to ensure accuracy.

Additionally, differences among treatment laterality—**BreastR**, **BreastL**, and **Bilat_Breast**—highlight the influence of anatomical and dosimetric complexity on GPR. Although right and left breast treatments generally maintain high GPR values, bilateral breast treatments exhibit greater variability, likely due to their inherently more complex nature.

Overall, the observed variability in GPR associated with MUs, heart and breast volumes, and treatment laterality underscores the need for robust predictive models. Such models can proactively identify potentially suboptimal plans, enhancing the efficiency and reliability of the PSQA process.

### 3.2 Models' performance on training dataset

The predictive performance of the trained machine learning models was evaluated using cross‐validation on the training dataset. The evaluation employed three key metrics: **mean squared error (MSE)**, **root mean squared error (RMSE)**, and **mean absolute error (MAE)**, along with their respective standard deviations to assess model stability. The results are summarized in Table 2.

**TABLE 2:** Mean squared error (MSE), root mean squared error (RMSE), and mean absolute error (MAE) for each model and their respective standard deviation obtained from testing dataset. (Hist gradient boosting regressor (HGBR), random forest regressor (RFR), extra trees regressor (ETR), gradient boosting regressor (GBR), linear regression (LR), Ada Boost regressor (ABR), and Multi‐layer perception regressor (MLPR).

Model	MSE (%)	Std MSE (%)	RMSE (%)	Std RMSE (%)	MAE (%)	Std MAE (%)
**HGR**	0.00464	0.00121	0.675	0.092	0.507	0.047
**RFR**	0.00445	0.00119	0.661	0.093	0.501	0.05
**ETR**	0.00434	0.00116	0.653	0.092	0.484	0.05
**GBR**	0.00431	0.00112	0.651	0.09	0.49	0.047
**LR**	0.00471	0.00130	0.679	0.097	0.503	0.067
**ABR**	0.00462	0.00121	0.674	0.092	0.509	0.052
**MLPR**	0.00563	0.00133	0.745	0.09	0.543	0.067

The **extra trees regressor (ETR)** and **gradient boosting regressor (GBR)** emerged as the top‐performing models, achieving the lowest mean MSE values (0.00431 and 0.00434, respectively) and RMSE values (0.651 and 0.653). These results indicate their strong ability to capture complex, non‐linear relationships in the data. Both models also demonstrated low variability, with standard deviations of 0.00112 (GBR) and 0.00117 (ETR), suggesting consistent performance across folds.

The **random forest regressor (RFR)** and **histogram‐based gradient boosting regressor (HGBR)** followed closely, offering a good balance between accuracy and interpretability. Although slightly less accurate, their performance remained within a clinically acceptable range.

In contrast, the **linear regression (LR)** model showed higher MSE (0.00471) and RMSE (0.680), along with greater variability. This reflects the model's limitations in capturing the non‐linear patterns inherent in complex VMAT treatment plans.

When comparing MAE values, the ETR, GBR, and RFR models again performed best, with mean absolute errors ranging from **0.480% to 0.495%**, and low standard deviations. These models are particularly effective at minimizing individual prediction errors, which is crucial for clinical reliability. Although LR and HGBR showed slightly higher MAEs (0.503 and 0.507, respectively), they still performed within acceptable limits. However, the higher variability observed in LR may pose challenges in clinical settings where prediction consistency is critical.

### 3.3 Models’ performance on validation dataset

To assess the generalizability of the trained models, their performance was evaluated on the validation dataset. Table 3 summarizes the results using the same metrics as in training: **MSE**, **RMSE**, and **MAE**.

**TABLE 3:** Mean squared error (MSE), Root mean squared error (RMSE), and mean absolute error (MAE) for each model obtained from testing dataset (Hist gradient boosting regressor (HGBR), random forest regressor (RFR), extra trees regressor (ETR), gradient boosting regressor (GBR), linear regression (LR), Ada Boost regressor (ABR), and Multi‐layer perception regressor (MLPR).

Model	MSE (%)	RMSE (%)	MAE (%)
**HGR**	0.0053	0.0731	0.5281
**RFR**	0.0051	7.1448	0.5124
**ETR**	0.0051	7.1476	0.5086
**GBR**	0.0052	0.0720	0.5108
**LR**	0.0053	0.0725	0.5240
**ABR**	0.0054	0.0737	0.5277
**MLPR**	0.0057	0.0756	0.5499

The **RFR** and **ETR** achieved the lowest MSEs among all models, confirming their effectiveness in minimizing prediction errors. These models also demonstrated strong MAE performance, with values of **0.5124%** (RFR) and **0.5086%** (ETR), indicating their ability to maintain low average prediction errors.

The **GBR** followed closely, with a slightly higher MSE (0.0052%) and MAE (0.5108%). However, it stood out with the lowest RMSE (0.0720%), suggesting superior handling of larger individual errors.

The **HGR** showed comparable performance to GBR in terms of MSE (0.0053%) but had a slightly higher MAE (0.5281%), indicating a modest increase in average prediction error.

The **LR** model delivered respectable results (MSE = 0.0053%, MAE = 0.5240%), but was outperformed by ensemble models in both accuracy and robustness. Similarly, the **ABR** produced slightly less favorable metrics (MSE = 0.0054%, MAE = 0.5277%), suggesting limitations in capturing complex data interactions.

The **MLPR** showed the weakest performance, with the highest MSE (0.0057%) and MAE (0.5499%). This may be due to suboptimal hyperparameter tuning or architectural limitations that hindered its ability to model the data effectively.

Overall, ensemble models—particularly **ETR**, **RFR**, and **GBR**—demonstrated the best performance on the validation dataset. Among them, **ETR** slightly outperformed the others in terms of MAE, making it a strong candidate for predicting GPR in pre‐treatment quality assurance for breast VMAT plans.

### 3.4 Comparison between two best models

**FIGURE 3:**
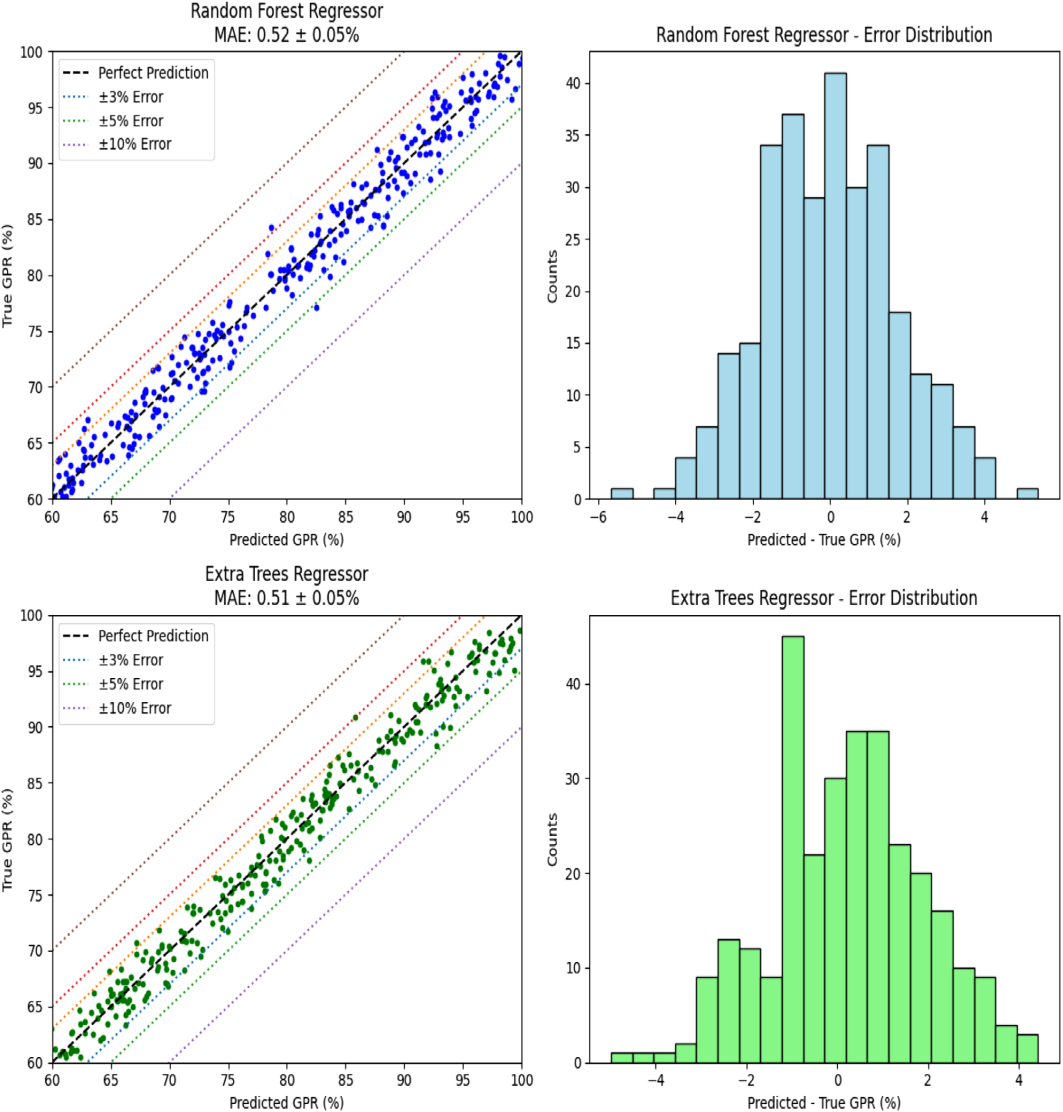
Performance comparison of the random forest regressor (RFR) and extra trees regressor (ETR) models. The graphs on the left show the agreement between predicted and actual values, while the histograms on the right illustrate the error distribution (difference between predicted and actual values).

This section presents a comparative analysis of the **RFR** and **ETR** models using graphical evaluation. Figure 3 illustrates the prediction quality of both models through concordance plots and error distribution histograms.

#### 3.4.1 Concordance analysis

The left panels of Figure 3 display concordance plots, which compare predicted versus actual GPR values. The black dashed line represents perfect prediction, while shaded bands indicate error margins of ± 3%, ± 5%, and ± 10%. Both RFR and ETR models show a high density of points near the perfect prediction line, confirming their strong predictive capabilities.

Notably, the ETR model demonstrates a tighter clustering of predictions within the ± 3% band, suggesting slightly superior precision. This indicates that ETR is more consistent in producing predictions that closely match actual GPR values.

#### 3.4.2 Error distribution analysis

The right panels of Figure 3 show histograms of prediction errors (actual—predicted GPR). Both models exhibit symmetrical distributions centered around zero, indicating no systematic bias in predictions. However, the ETR model's error distribution is narrower, reflecting better control over large deviations and a more stable prediction pattern.

#### 3.4.3 Performance summary

- **MAE** values for RFR and ETR are **0.52%** and **0.51%**, respectively demonstrating nearly equivalent average prediction accuracy.
- The **ETR model** shows a slight edge in minimizing large errors and maintaining tighter prediction intervals.
- Both models exhibit minimal variability in error patterns, reinforcing their reliability for clinical use.

The comparative analysis confirms that both RFR and ETR are highly effective for predicting GPR in breast VMAT pre‐treatment quality assurance. However, the **ETR model** demonstrates a minor but meaningful advantage in terms of accuracy and stability. These findings support the use of ensemble learning techniques—particularly ETR—as robust tools for automating and enhancing PSQA workflows in clinical settings.

## 4 DISCUSSION

The prediction of GPR is a critical component of PSQA in radiation therapy, particularly for breast cancer treatments using VMAT. This study explored the application of ML models to predict GPR, aiming to streamline PSQA workflows while maintaining high standards of treatment accuracy and safety.

To our knowledge, this is the first study to specifically address GPR prediction for breast cancer PSQA in VMAT. As such, our findings are compared with existing literature that primarily focuses on multi‐site or general radiotherapy datasets.

In our analysis, the **ETR**, **RFR**, and  **GBR** models demonstrated superior performance, achieving mean absolute errors (MAEs) of **0.51%**, **0.52%**, and **0.51%**, respectively. These results are notably better than those reported in prior studies. For instance, Bin et al.^31^ used a Random Forest model on 269 IMRT plans and reported an MAE of **1.81%** and RMSE of **2.14%**, with a correlation coefficient of **0.72**. Similarly, Wall et al.^32^ evaluated 500 multi‐anatomical VMAT plans using various models and reported an MAE of **3.75%** for GPR prediction under 3%/3 mm criteria.

Zhu et al.^33^ applied ensemble models to 213 IMRT plans and achieved a mean error of **0.78%** under 3%/2 mm criteria, further supporting the effectiveness of ensemble methods in modeling complex radiotherapy data. These comparisons underscore the strength of our approach, particularly the benefit of focusing on a single anatomical site, which likely contributed to the improved model accuracy and robustness.

Our **LR** model yielded an MAE of **0.524%**, slightly higher than the ensemble models. This aligns with findings by Hirashima et al.,^34^ who noted that while linear models offer interpretability, they often fall short in capturing the non‐linear relationships inherent in radiotherapy data. Nonetheless, LR remains valuable in contexts where model transparency is essential, especially when geometric features like irradiated volumes play a significant role.^35^

The **MLPR** showed a slightly higher MAE of **0.549%**, which may be attributed to suboptimal hyperparameter tuning or the limited dataset size. This observation is consistent with Tamori et al.,^36^ who emphasized that neural networks require careful optimization and larger datasets to outperform ensemble models.

Overall, our results are consistent with and, in many cases, outperform those reported in the literature. The ETR, RFR, and GBR models stand out as optimal choices for GPR prediction due to their accuracy, robustness, and ability to model complex relationships. Although neural models showed potential, further optimization is needed. Linear models, though less accurate, remain useful for applications requiring interpretability.

A key limitation of this study is its reliance on retrospective data from a single institution. Future work will focus on external validation using datasets from additional centers to assess generalizability. Moreover, clinical integration could be achieved by embedding the predictive model into the treatment planning system or a dedicated QA triage dashboard, enabling real‐time feedback during plan development.

## 5 CONCLUSIONS

This study demonstrates the potential of machine learning ML models to enhance PSQA in VMAT for breast cancer treatment. Among the models evaluated, the **ETR**, **RFR**, and **GBR** consistently delivered the most accurate predictions of GPR, with minimal error variability.

These ensemble models offer a promising alternative to traditional, resource‐intensive PSQA procedures by enabling early identification of treatment plans that may require additional verification. Their predictive accuracy and robustness make them strong candidates for integration into clinical workflows.

Although simpler models such as linear regression provided acceptable performance, they were outperformed by ensemble methods in handling the complex, non‐linear relationships inherent in VMAT planning. The slightly lower performance of neural network models suggests that further optimization—particularly in hyperparameter tuning and dataset expansion—is needed to fully leverage their potential.

Looking ahead, integrating ML‐based GPR prediction into clinical systems—either within treatment planning software or through dedicated QA dashboards—could significantly streamline QA processes. This would not only improve operational efficiency but also enhance the consistency and safety of radiotherapy delivery, ultimately contributing to better patient outcomes.

## AUTHOR CONTRIBUTIONS

Francis C. Djoumessi Zamo conceptualized the project, was involved in data collection, data analysis, drafting, and reviewing of the manuscript. Anthony Colliaux was involved in data collection, data analysis, drafting, and reviewing of the manuscript. Valérie Blot‐Lafond was involved in data collection, data analysis, drafting, and reviewing of the manuscript. Prof. Ndontchueng Moyo conceptualized the project, data analysis, drafting, and reviewing of the manuscript. Christopher Njeh conceptualized the project, data analysis, drafting, and reviewing of the manuscript.

## CONFLICT OF INTEREST STATEMENT

The authors declare no conflict of interest.
